# Etiology of low testosterone levels in male patients with severe sepsis requiring mechanical ventilation

**DOI:** 10.1186/cc12386

**Published:** 2013-03-19

**Authors:** A Bech, H Van Leeuwen, H De Boer

**Affiliations:** 1 Rijnstate Hospital, Arnhem, the Netherlands

## Introduction

Low testosterone levels are frequently found in critically ill male patients. The etiology and clinical significance is still poorly understood. In the present study we have investigated the kinetics and pathophysiology of altered gonadal hormone synthesis in male patients with severe sepsis and respiratory failure.

## Methods

All male patients with severe sepsis and respiratory failure who were admitted to the ICU of a large teaching hospital in the Netherlands between September 2011 and June 2012 were included. Steroid hormone levels were measured on days 1, 3 and 7.

## Results

**Figure 1:**
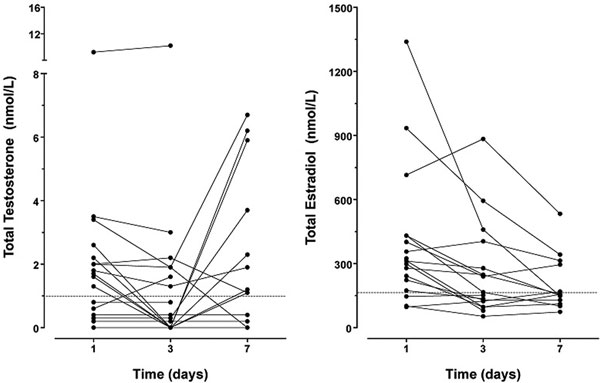


**Figure 2:**
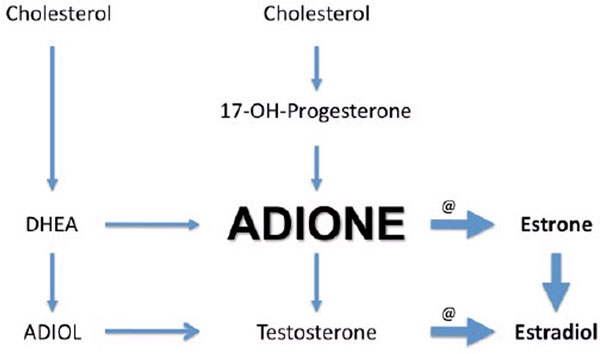


In total, 18 patients were included. The mean age was 69 ± 2 years, mean weight 76 ± 2 kg, APACHE II score 23 ± 2 and most patients suffered from pneumosepsis. On the first days of intubation, total and free testosterone levels were extremely low in most patients and remained low during the first week (Figure 1). 17β-Estradiol levels were elevated on day 1 and decreased during the first week. LH and FSH levels were inappropriately low. All lipoprotein fractions and their apo-proteins were reduced as well as 17-OH-progesterone, DHEA and DHEAS. In contrast, androstenedione (adione) levels were elevated. This suggests preferential and stimulated synthesis of androstenedione (Figure 2). The high 17β-estradiol levels indicate that androstenedione is shunted into the estrogen pathway, a process that requires high aromatase activity. The high estradiol/total testosterone ratio supports this conclusion.

## Conclusion

Hyperestrogenic hypotestosteronemia is a frequent finding in the acute phase of severe sepsis in male patients with respiratory failure. It is suggested to be caused by decreased androgen production and shunting of androgen to estrogen synthesis as a result of increased aromatase activity. The clinical relevance of gonadal hormone substitution needs further study.

